# Reply to Behnam and Klein: Potential role of the His-tag in C-terminal His-tagged SARS-CoV-2 main protease

**DOI:** 10.1073/pnas.2108209118

**Published:** 2021-09-02

**Authors:** Zhe Li, Runduo Liu, Chang-Guo Zhan, Xin Wang, Hai-Bin Luo

**Affiliations:** ^a^Guangdong Provincial Key Laboratory of New Drug Design and Evaluation, School of Pharmaceutical Sciences, Sun Yat-Sen University, Guangzhou 510006, People’s Republic of China;; ^b^Molecular Modeling and Biopharmaceutical Center, College of Pharmacy, University of Kentucky, Lexington, KY 40536;; ^c^Department of Pharmaceutical Sciences, College of Pharmacy, University of Kentucky, Lexington, KY 40536;; ^d^Center for Innovative Marine Drug Screening & Evaluation, School of Medicine and Pharmacy, Ocean University of China, Qingdao 266100, China;; ^e^Marine Biomedical Research Institute of Qingdao, Qingdao 266100, China;; ^f^Key Laboratory of Tropical Biological Resources of Ministry of Education, School of Life and Pharmaceutical Sciences, Hainan University, Haikou 570228, China

Behnam and Klein (1) carried out in vitro assays on two severe acute respiratory syndrome coronavirus 2 (SARS-CoV-2) main protease (M^pro^) inhibitors, including atazanavir reported by us (2), using an M^pro^ protein with C-terminal His-tag under various assay conditions, and find that both the Michaelis−Menten constant (*K*_m_) and inhibitory activity (IC_50_) are sensitive to the assay conditions. It is interesting to examine whether the assay conditions significantly affect the activities of an enzyme and its inhibitors. On the other hand, Behnam and Klein (1) do not account for possible effects of the C-terminal His-tag on the *K*_m_ and IC_50_, despite the fact that the tag-free M^pro^ (without any N- or C-terminal tag) was used in our reported assays (2), whereas the C-terminal His-tagged M^pro^ was used in their assays (1).

As discussed recently (3), both the N and C termini of M^pro^ are close to the active-site cavity, according to available X-ray crystal structures. Thus, a His-tag on the N or C terminus could interfere in M^pro^ binding with a ligand (substrate or inhibitor). Depicted in Fig. 1 is a modeled structure of the C-terminal His-tagged M^pro^ protein. The modeling started from an available X-ray crystal structure (Protein Data Bank ID code 6M2N) (4). Notably, the His-tag was outside of the M^pro^ active site in the initial structure (Fig. 1*A*) built by using the Prime module (5, 6) of the Schrodinger 2013 software. After 50-ns molecular dynamics (MD) simulations, the His-tag moved to the M^pro^ active site (Fig. 1*B*), suggesting that the His-tag added to the C terminus may compete with the substrate/inhibitor for binding. For this reason, the C-terminal His-tag could lower the binding affinity of a given ligand (substrate or inhibitor) with M^pro^. In fact, it has been reported that *K*_m_ = 1.41 μM for the tag-free M^pro^ (3) and *K*_m_ = 28.2 μM for the C-terminal His-tagged M^pro^ (7), suggesting that the C-terminal His-tag may lower the binding affinity with the substrate by ∼20-fold. The remarkable difference explains why the IC_50_ values determined by Behnam and Klein (1) and Ma and Wang (8) using C-terminal His-tagged M^pro^ are significantly larger than the corresponding IC_50_ values determined by us using the tag-free M^pro^ (2). It is also possible that the interaction between the His-tag and the active site is affected by the assay conditions. Hence, the results described by Behnam and Klein (1) are not surprising based on the structural information about the His-tagged M^pro^ protein discussed above.

**Fig. 1.**
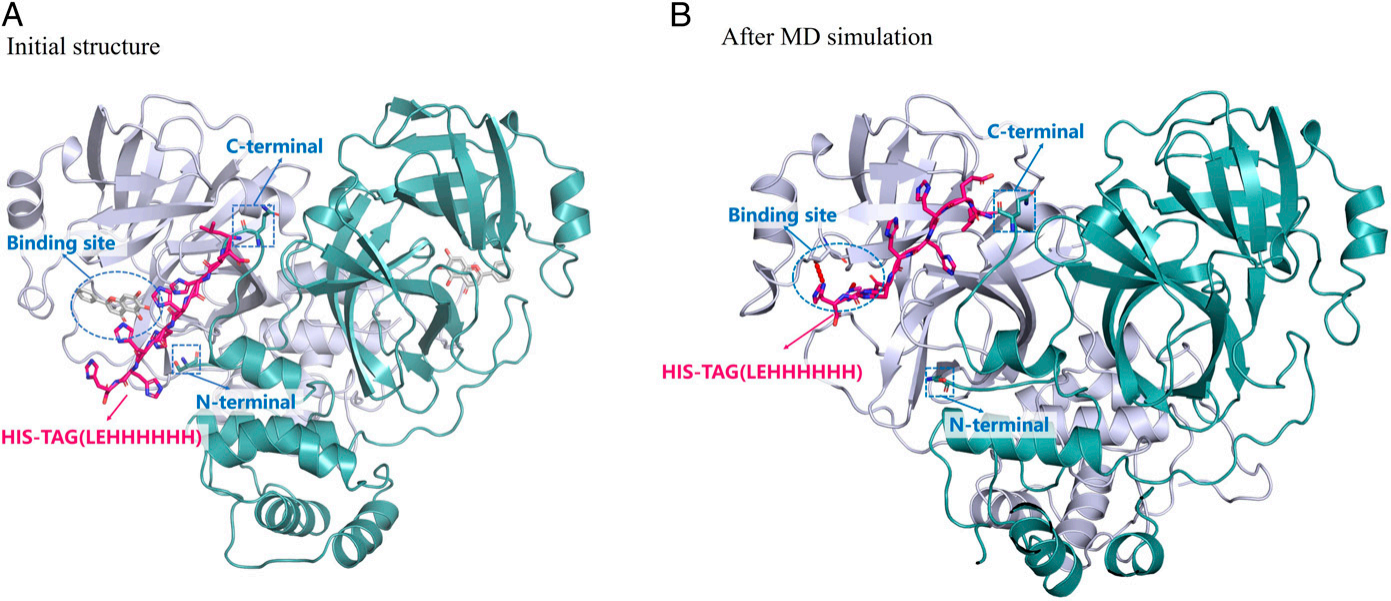
Structure of the M^pro^ protein with His-tag (LEHHHHHH) added to the C terminus. (*A*) Initial structure of the C-terminal His-tagged M^pro^ protein with a ligand bound in the active site. (*B*) The last snapshot of the MD-simulated C-terminal His-tagged M^pro^ protein structure (in which the ligand in the active site was removed before the MD simulation) in a TIP3P water box. The system, after energy minimization, was first heated to 300 K in a 50-ps MD simulation (with NVT) followed by 50-ps MD simulation [with NPT, i.e., the constant amount of substance (N), pressure (P), and temperature (T)] for equilibration. Finally, a 50-ns MD simulation (with NPT) was performed without any restraints for the system.

## Change History

September 8, 2021: The licensing has been updated.
